# Investigation on tribological behaviors of biodegradable pure Zn and Zn-X (Li, Cu, Ge) binary alloys

**DOI:** 10.1007/s10856-021-06625-4

**Published:** 2021-12-04

**Authors:** Huafang Li, Jinyan Huang, Peng Zhang, Qi Zhang

**Affiliations:** 1 School of Materials Science and Engineering, University of Science and Technology Beijing, Beijing, 100083 China; 2 State Key Laboratory for Advanced Metals and Materials, University of Science and Technology Beijing, Beijing, 100083 China

## Abstract

As a potential biodegradable implant material, zinc (Zn) alloys have attracted increasing attention due to their good biocompatibility and moderate degradation rate. Zn and its alloys are expected to become candidate materials for medical devices. The metals implanted in the human body will inevitably undergo friction in the human body before it is completely degraded. Friction and wear are essential factors which may cause medical devices’ service failure. However, there are still few studies on the friction and wear properties of biodegradable Zn-based alloys in the human body, and most studies just focus on the mechanical properties, degradation properties and biocompatibility of the alloys. Thus, it is crucial to study the friction and wear properties of Zn and its alloys. In the present work, we investigated the tribological properties of biodegradable pure Zn and Zn-X (Li, Cu, Ge) alloys. Our study found that under simulated body fluid and dry friction conditions, the addition of alloying elements Li and Cu can improve the friction properties of Zn. Among the four metals, Zn-0.5Li alloy has the lowest friction coefficient and the best wear resistance. Hank’s solution has lubricating and corrosive effects. That is to say, when the alloy is rubbed in Hank’s solution, it can not only be protected by the lubrication of the solution, but also tribocorrosion will occur as well.

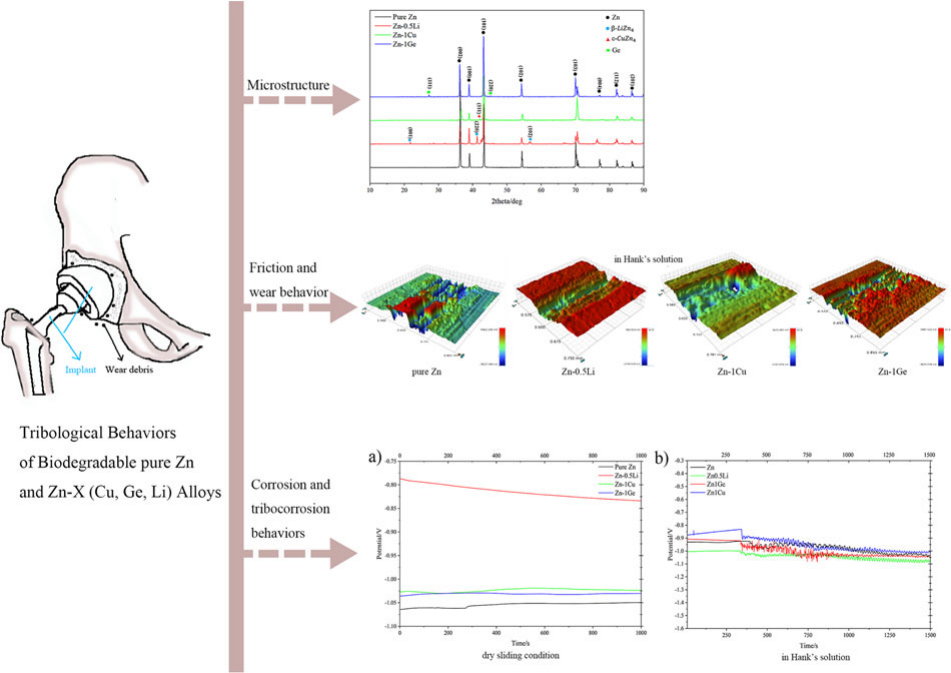

## Introduction

Biomedical metallic materials include traditional bioinert metallic materials for load-bearing and biodegradable metals. Biodegradable metals (BMs) are ideal temporary implant materials because of their good degradability and biocompatibility [1]. Due to good mechanical properties and corrosion resistance, many traditional bioinert metals (such as Ni-Ti, Ti-Nb, Ti-6Al-4V, etc.) have been used in biomedical applications for bone implants and heart valves [2]. However, traditional bioinert metals that are permanently implanted in the body require a second operation to be removed from the patient after implantation. BMs degrade gradually after implantation and promote complete healing of local tissues. It avoids the need for a second removal surgery, thereby reducing patient’s medical costs and physical pains [1, 3]. Iron (Fe)- based and magnesium (Mg)-based biodegradable metals are the two mostly studied metals in biodegradable metals [4–9]. Compared with Fe- based and Mg-based alloys, Zn-based alloys have a more appropriate corrosion rate and will not generate hydrogen during the degradation process [10]. As a new type of biodegradable metal, Zn-based alloys have attracted increasing attentions due to their moderate corrosion rate, good biocompatibility, and physical properties [11, 12]. In recent years, Zn-based alloys have appeared in orthopedic equipment, cardiovascular stents and other medical fields [10]. Pure zinc has poor mechanical properties (the ultimate stregnth of as-cast pure Zn is <50 MPa), which cannot meet the requirements of biomedical applications (yield strength>200 MPa, ultimate tensile strength>300 MPa, elongation > 15–18%) [13]. Pure zinc can improve its mechanical properties through alloying treatment (such as adding elements Li [14], Mg [15], Ca [16], Ge [17] and Cu [18]).

In the human body, a degree of micromotion of the bone-implant interface is unavoidable [19, 20]. The wear of materials in the body will produce a series of adverse effects. Wear of materials produces abrasive particles, which can damage cells tissue and cause carcinogenic responses [21–23]. It can also lead to osteolysis, inflammation and other adverse reactions [24, 25]. As an implant material, the inflammation and bone absorption around the material caused by friction with the human joints after implantation in the human body are the main reasons for the failure of implant surgery [26]. Therefore, the implant material must have suitable tribological properties, which can reduce the generation of wear debris and prevent inflammation and bone resorption.

In the past, researchers have studied the friction and wear behavior of bioinert metallic materials (such as Ti-based alloys [27–30], Co-based alloys [31, 32], stainless steel [33] and Ti-Ni shape memory alloys [34]). However, there are few studies on the friction and wear properties of degradable Zn alloys at present, which basically focus on their general mechanical properties (strength, elongation, etc.) and corrosion properties [20]. Thus, it is crucial to study the friction and wear properties of Zn-based alloys.

The aim of the present work is to investigate the tribological behavior of pure Zn and Zn-based alloys after alloying of Zn with different alloying elements Li, Cu, and Ge. Besides, the wear resistance of different Zn-based alloys is compared and the corrosion and tribocorrosion behaviors of pure Zn and Zn-based alloys in simulated body fluids are studied as well.

## Materials and methods

### Material preparation

The materials used in this experiment are pure Zn, Zn-0.5 (wt.%) Li, Zn-1(wt.%) Cu and Zn-1(wt.%) Ge. High purity Zn(99.996%), Li(99.95%), Cu(99.99%), Ge(99.99%) were vacuum melted at 700 °C to obtain ingots. The as-cast Zn, Zn-0.5Li, Zn-1Cu and Zn-1Ge ingots were further undergone the hot-rolled treatments to get the as-rolled samples. For hot-rolling, plates were rolled down to 1.5 mm at a rolling reduction in thickness of 0.2 mm per pass after preheating at 300 °C for 10 min. The specimens with geometric size of 20 mm × 5 mm × 1.5 mm was obtained from the hot-rolled plate via electrical discharge wire - cutting. The specimens were polished with 400#, 800#, 1500#, 2500# and 5000# sandpaper, respectively, followed by ultrasonically cleaned in acetone, absolute ethanol and distilled water for 10 min and then dried in air.

### Microstructure characterization

The phase analysis of the sample was carried out by multifunction X-ray diffractometer (XRD, TTRIII, Rigaku Corporation, Japan, Cu K_α_). The parameters are as follows: the scanning scope:10°~90°; the scanning rate is 10°/min; the step size is 0.02°; the voltage is 40 kV and the current is 300 mA.

MDI JADE 5 analysis software was used to analyze the obtained XRD data to determine the phase composition of the samples.

### Friction and wear testing

The friction and wear tests of pure Zn, Zn-0.5Li, Zn-1Cu and Zn-1Ge under dry sliding and Hank’s solution simulated body fluid conditions were conducted in a friction tester(tribometer) (UMT, Bruker, Germany). The test was carried out under a wear load of 1 N, a testing time of 30 min, a sliding speed of 1 mm/s, and a friction pair of Si_3_N_4_.

The coefficient of friction (COF) is calculated according to Eq. (1):

1 $$COF = \frac{{F_X}}{{F_Z}}$$

where F_Z_ is the normal force and F_X_ is the tangential friction force, both of which can be measured by the friction and wear testing machine (UMT).

After the test, evaluation of the microstructure and chemical compositions of the phases of the alloy samples was carried out using a scanning electron microscopy (SEM, EVO18, ZEISS, Germany) with an energy dispersive spectrometer (EDS, Quantax, Bruker, Germany).

A white light interference microscope (ContourGT, Bruker, Germany) was used to observe the three-dimensional morphology of the samples after the friction and wear test, and the width and depth of the wear tracks were measured. Pure Zn and Zn alloys, which were subjected to frictional experiments in Hank’s solution, were soaked in ammonium chloride solution (100 g/L NH_4_Cl) at 70 °C for 5 min to remove the corrosion products according to ISO 8407:2021 [35].

### Electrochemical testing

An electrochemical working station (CHI 630, CH Instruments, Shanghai) was used for the electrochemical tests at 37 °C in Hank’s solution. A platinum electrode was set as the auxiliary electrode, a saturated silver chloride electrode as the reference electrode, and the specimens as the working electrode, respectively. The polarization curves and OCP curves were obtained by electrochemical testing. Polarization curves were recorded over the potential range of −1.38 V to +0.50 V at a scanning rate of 0.005 mV/s.

## Results

### Microstructure characterization

**Fig. 1:**
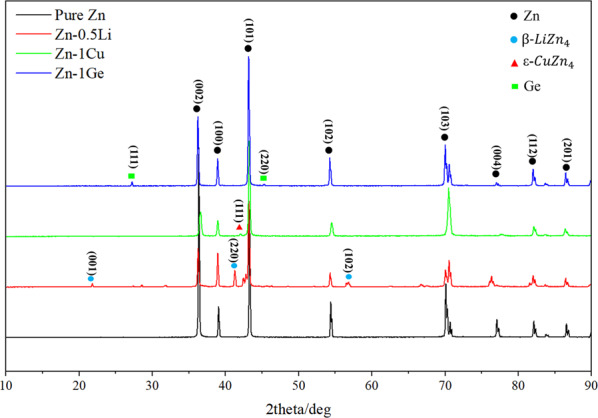
XRD patterns of Pure Zn, Zn-0.5Li, Zn-1Cu, Zn-1Ge.

Figure 1 shows the XRD patterns of the pure Zn, Zn-0.5Li, Zn-1Cu and Zn-1Ge. Pure Zn is a metal with a hexagonal close packed structure (hcp). The XRD results showed that after the alloying treatment of pure zinc, new phases appeared in the alloys in addition to the matrix Zn with a hcp structure. Zn-1Ge consisted of Zn and Ge phase. β-LiZn_4_ phase was found in Zn-0.5Li and ε-CuZn_4_ was found in Zn-1Cu.

### Friction and wear behavior

**Fig. 2:**
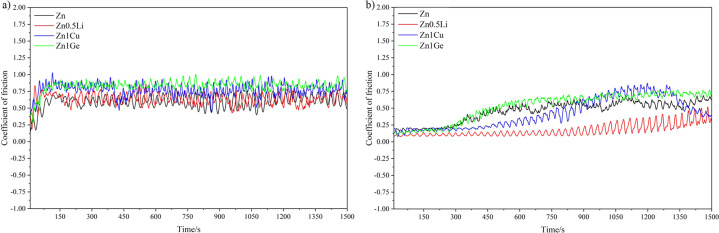
Coefficient of friction vs sliding time of four metals: (**a**) under dry sliding condition; (**b**) in simulated body fluids (Hank’s solution).

Figure 2 shows the trend of friction coefficient of pure Zn and Zn alloys under dry sliding (Fig. 2a) and Hank’s solution (Fig. 2b) condition. It can be seen that coefficient of friction of the four metals presented some fluctuation during sliding. Among the four samples, the curve of the friction coefficient of Zn-0.5Li alloy is the lowest. Under dry sliding friction condition, the friction coefficient of the sample increases gradually at the beginning of sliding, and then fluctuates in a certain range. The coefficient of friction curve of Zn-1Ge alloy is relatively stable under dry sliding friction condition. The coefficient of friction of pure Zn and Zn-0.5Li alloys changes sharply, and the fluctuation increases with time. In Hank’s solution, within 300 s of the beginning of friction, the value of the friction coefficient of the samples is small, and the friction coefficient fluctuates in a small range between 0 and 0.25 (probably due to the lubrication effect of Hank’s solution). Subsequently, the value of the friction coefficient increased with the increase of the test time in Hank’s solution, and the fluctuation range of the friction coefficient gradually increased. In a long-term friction and wear test, the friction coefficient under the lubrication condition of Hank’s solution is lower than the friction coefficient under the dry sliding condition. However, the stability of the coefficient of friction in Hank’s solution is less than that under dry sliding friction conditions.

In order to more accurately compare the coefficient of friction (COF) of the four samples, the mean values and standard deviations of the COF of the four samples were calculated (as shown in Table 1). Under the dry sliding condition, the COF of the four metals from large to small are as follows: pure Zn > Zn-1Ge > Zn-1Cu > Zn-0.5Li. In the Hank’s solution, the COF of the four metals from large to small are as follows: Zn-1Geå pure Znå Zn-1Cuå Zn-0.5Li. By comparing the friction and wear coefficients in the dry environment, it can be seen that the coefficient of friction of pure Zn, Zn-0.5Li, and Zn-1Cu decrease substantially in the simulated body fluid environment. In contrast, for Zn-1Ge, the coefficient of friction in Hank’s solution is slightly increased. This is due to the relatively low corrosion resistance of Zn-1Ge alloy in Hank’s solution (which can be confirmed in the electrochemical performance data below). It was found that the COF of pure Zn and Zn-1Ge is larger and the wear resistance is poor, while the COF of Zn-1Cu and Zn-0.5Li is smaller and the wear resistance is good.

**Table 1:** The Mean coefficient of friction (COF) of four metals under dry sliding condition and in Hank’s solution.

Dry sliding		Hank’s solution	
Metals	COF	Metals	COF
Pure Zn	0.626 ± 0.021	Pure Zn	0.447 ± 0.126
Zn-0.5Li	0.405 ± 0.029	Zn-0.5Li	0.248 ± 0.050
Zn-1Cu	0.611 ± 0.052	Zn-1Cu	0.404 ± 0.044
Zn-1Ge	0.622 ± 0.003	Zn-1Ge	0.627 ± 0.104

**Fig. 3:**
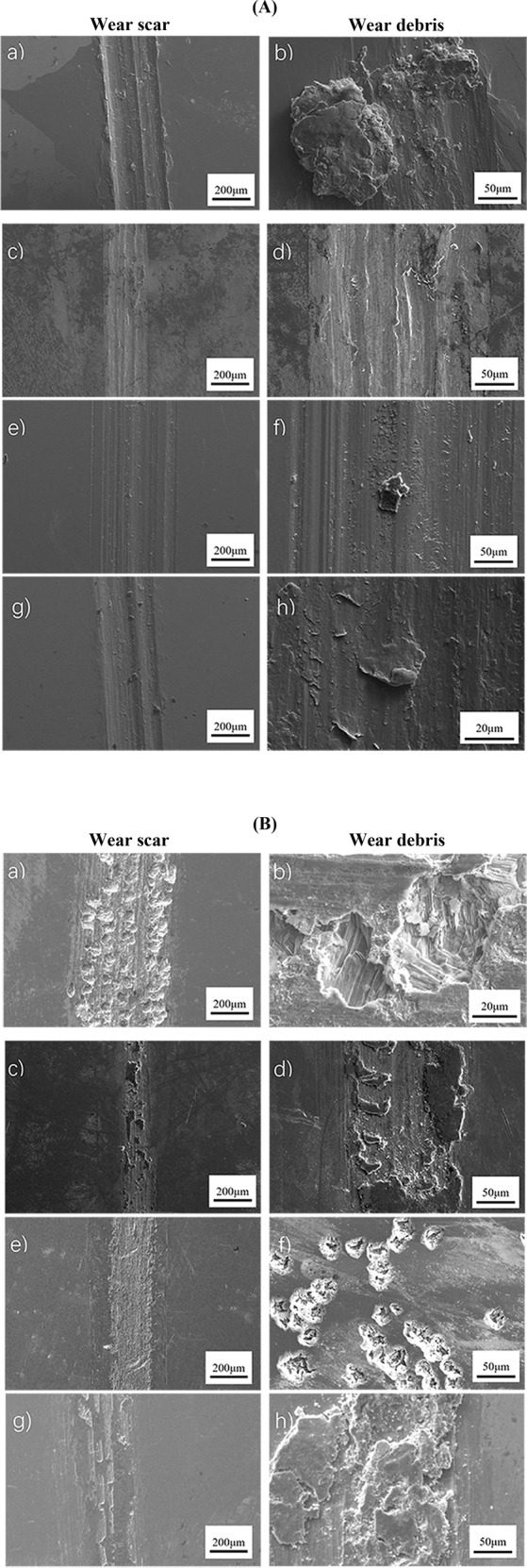
**A** SEM micrograph of wear scar and wear debris of four samples under dry sliding condition: (**a**), (**b**) Pure Zn; (**c**), (**d**) Zn-0.5Li; (**e**), (**f**) Zn-1Cu; (**g**), (**h**) Zn-1Ge; (**B**) SEM micrograph of wear scar and wear debris of four samples in Hank’s solution: (**a**), (**b**) Pure Zn; (**c**), (**d**) Zn-0.5Li; (**e**), (**f**) Zn-1Cu; (**g**), (**h**) Zn-1Ge.

**Fig. 4:**
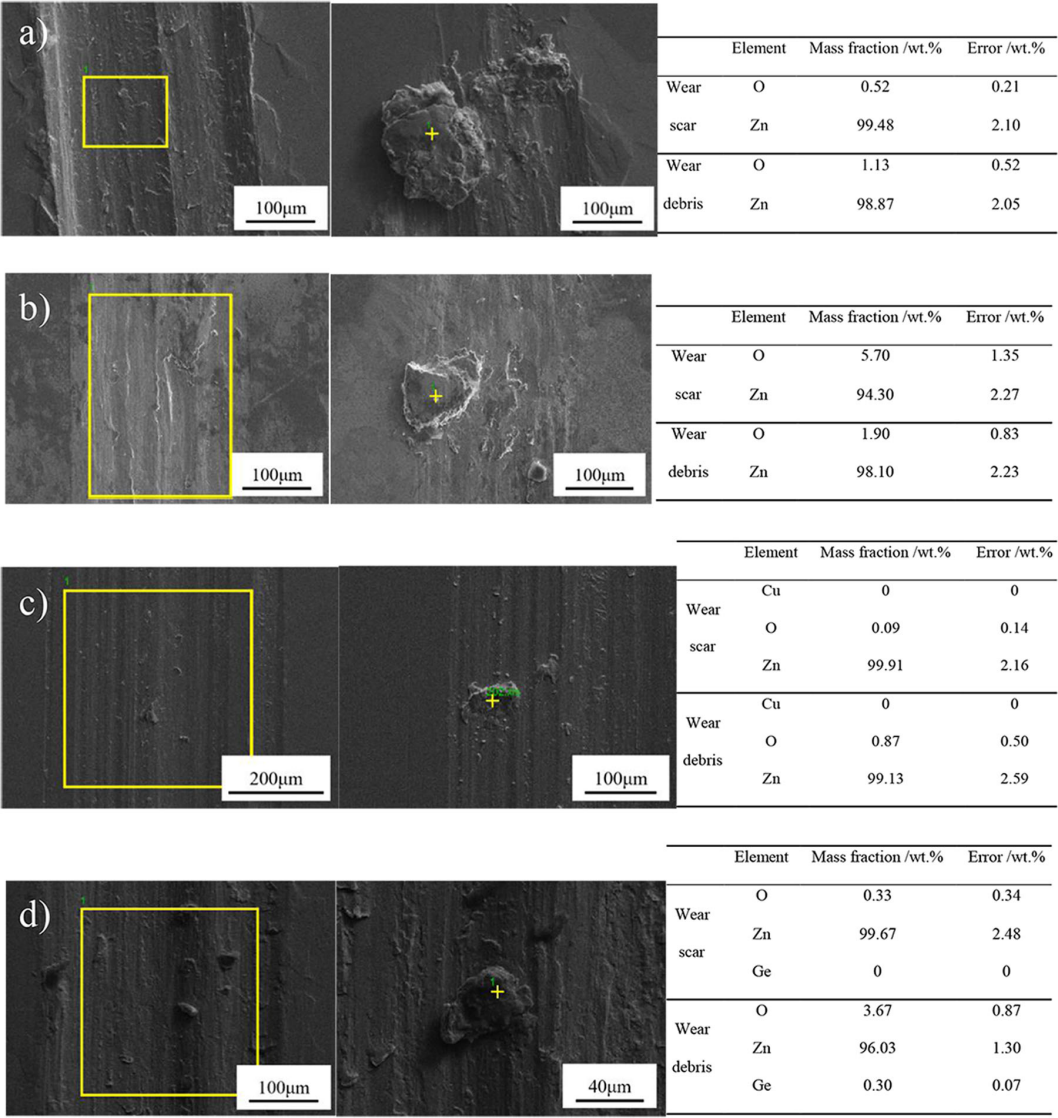
Elemental distributions of wear scars and debris of samples tested under dry sliding condition analyzed by EDS: (**a**) pure Zn; (**b**) Zn-0.5Li; (**c**) Zn-1Cu; and (**d**) Zn-1Ge.

Fig. 3 shows the surface morphology characteristics of pure Zn, Zn-0.5Li, Zn-1Cu and Zn-1Ge samples. The wear tracks of the alloys show the presence of typical wear features including wear scars, ploughing grooves and delamination. There are many grooves parallel to the sliding direction under dry sliding condition (See Fig. 3A). Among the four samples, the pure Zn has the most wear debris in the field of view, and the size of the particles is larger; Zn-0.5Li alloy shows signs of delamination and has the least amount of wear debris, accompanied by a small amount of oxide, which can be verified by EDS analysis (Fig. 4b).

**Fig. 5:**
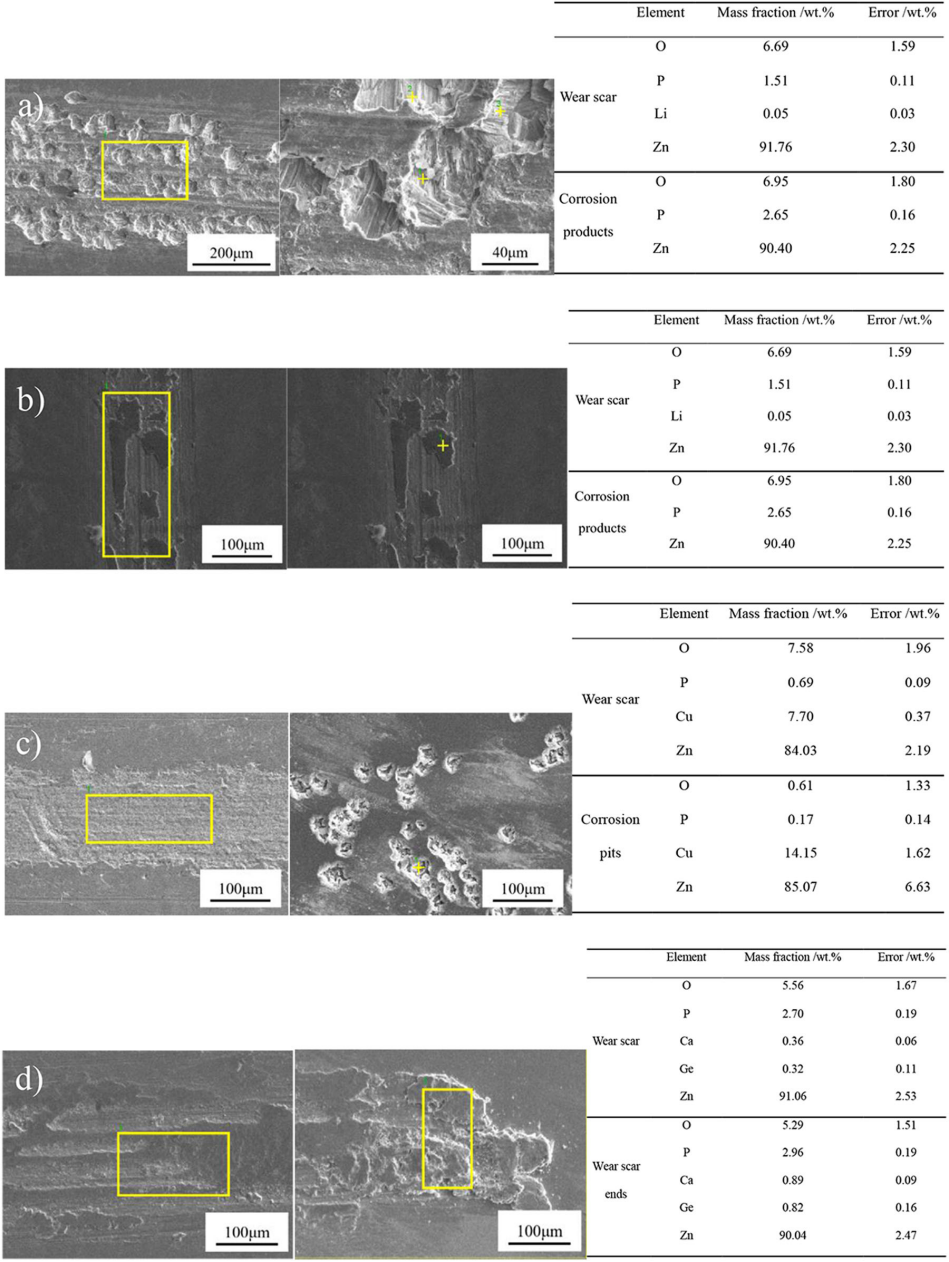
Elemental distributions of wear scars and debris of samples tested under Hank’s solution condition analyzed by EDS: (**a**) pure Zn; (**b**) Zn-0.5Li; (**c**) Zn-1Cu; and (**d**) Zn-1Ge.

Figure 3B shows the characteristics of surface morphology of pure Zn, Zn-0.5Li, Zn-1Cu and Zn-1Ge samples in the Hank’s solution simulated body fluid environment. The surface of pure Zn in this environment is rougher than that in dry environment, and corrosion pits appear on the surface. There is no obvious corrosion pit in Zn-0.5Li alloy, but there is accumulation of corrosion products on the surface. There are almost no corrosion pits in the abrasion marks of Zn-1Cu alloy, but there are more intensive corrosion pits in the unrubbed area of the sample. Corrosion products accumulate in the wear scar of Zn-1Ge. Compared with pure Zn, the abrasion marks of the other three Zn-based alloys are narrower and the corrosion is lighter. Compared with the friction and wear in the dry environment, the surface of the four samples in the simulated body fluid environment was almost free of lumpy debris. However, at high magnification, many very fine particles can be seen distributed around surface bulges and corrosion pits. These surface bulge may be part of the corrosion products produced by the metal in the simulated body fluid environment, and their content and composition are given in the EDS energy spectrum analysis results (Fig. 5).

Under dry sliding condition, the results of EDS analysis of pure Zn, Zn-0.5Li, Zn-1Cu and Zn-1Ge alloys are given in Fig. 4. According to the EDS analysis results, among the three samples (pure Zn, Zn-1Cu alloy, Zn-1Ge alloy) that can produce wear debris, the content of other elements except Zn (such as element O) in the wear debris is higher than that in the wear scar. However, the content of O in the wear debris of Zn-0.5Li is lower than that in the wear scar.

EDS analysis results of the surface of pure Zn and Zn alloys after friction and wear in Hank’s solution are shown in the figure below (See Fig. 5). No element Li was detected in the corrosion products of Zn-0.5Li alloy, while the contents of element O and P were higher than those in the wear scars. Compared with the dry sliding conditions, the element O content of all pure Zn and Zn alloys in the simulated body fluid environment increased. For Zn-0.5Li alloy, Zn-1Cu alloy and Zn-1Ge alloy, the contents of alloying elements (Li, Cu, Ge) are also increased. EDS analysis showed that the corrosion products were mainly composed of Zn, O, P and Ca elements, which might be the zinc (calcium) phosphates and zinc hydroxide.

**Fig. 6:**
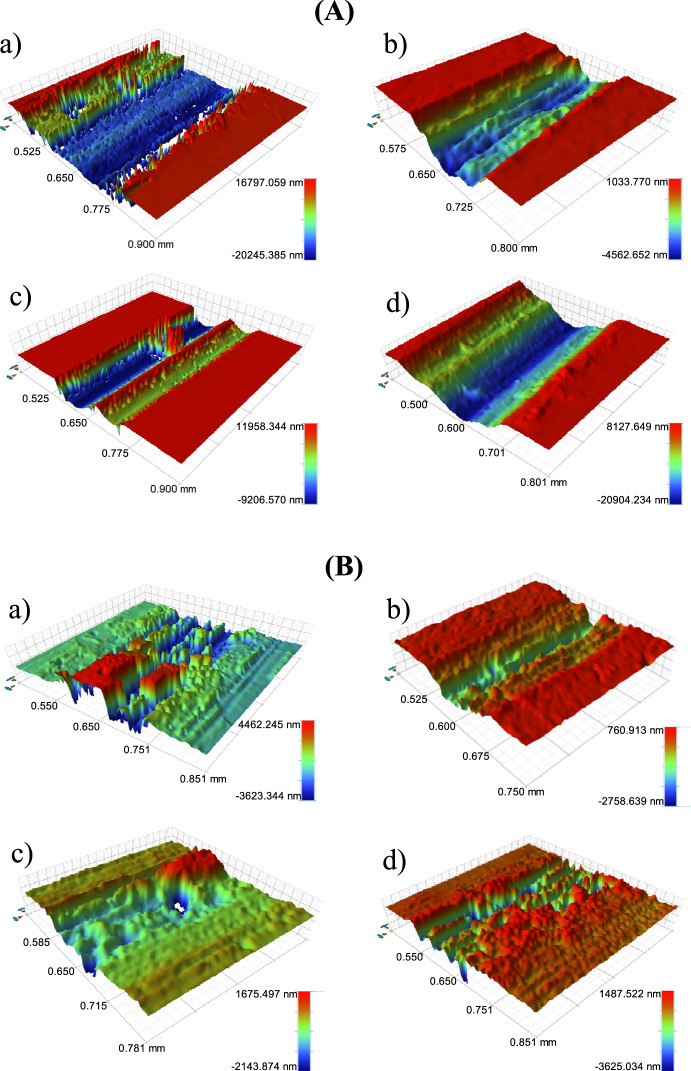
**A** Surface topographies of wear tracks tested under dry sliding condition: (**a**) pure Zn; (**b**) Zn-0.5Li; (**c**) Zn-1Cu; and (**d**) Zn-1Ge; (**B**) Surface topographies of wear tracks tested under Hanks’ solution condition: (**a**) Pure Zn; (**b**) Zn-0.5Li; (**c**) Zn-1Cu; and (**d**) Zn-1Ge.

The white light interference microscope was used to analyze the 3D morphologies of the wear tracks under dry sliding conditions and in the simulated body fluid environment, respectively (Fig. 6). It can be seen that all samples had obvious furrows and severe plastic deformation. Among the four samples, under dry sliding conditions, the wear scar depth and width of pure Zn and Zn-1Ge are large, and the wear is severe; the wear traces of Zn-1Cu and Zn-0.5Li alloys are shallow and narrow, and the wear is the lightest. In Hank’s solution, the wear width of Zn-0.5Li was narrower and the unworn area was smoother. The Zn-1Cu wear mark width is wider, but the wear mark is shallow. In addition, Zn-1Ge has the roughest surface.

**Fig. 7:**
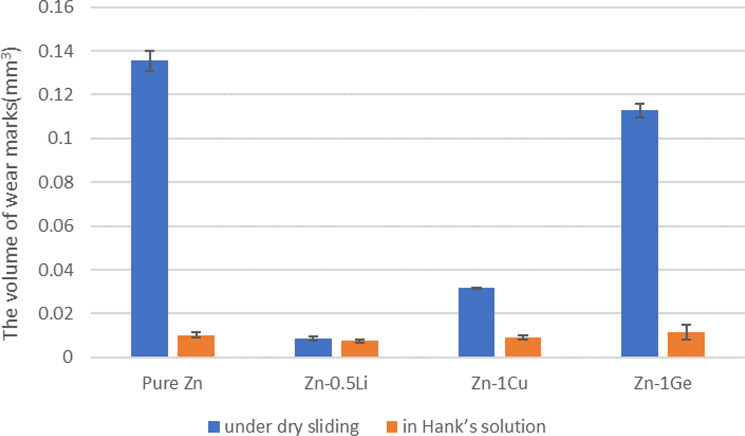
Volume of wear marks of the samples under dry sliding and in Hank’s solution.

The depth and width of wear marks of pure Zn and Zn alloys were multiplied to obtain the volume sizes of the wear marks (set the length of wear marks as 15 mm), as shown in Fig. 7. It can be seen from Fig. 7 that the sequence of wear mark volume of Zn alloys from large to small is: Zn-1Ge > Zn-1Cu > Zn-0.5Li. The addition of alloying elements (except for elements Ge) improves the wear resistance of pure Zn in simulated body fluids. Comparing the three Zn-based alloys (Zn-0.5Li, Zn-1Cu, Zn-1Ge), it is found that the alloy volume loss under the air condition is greater than that under the simulated body fluid (Hank’s solution) condition.

### Corrosion and tribocorrosion behavior

**Fig. 8:**
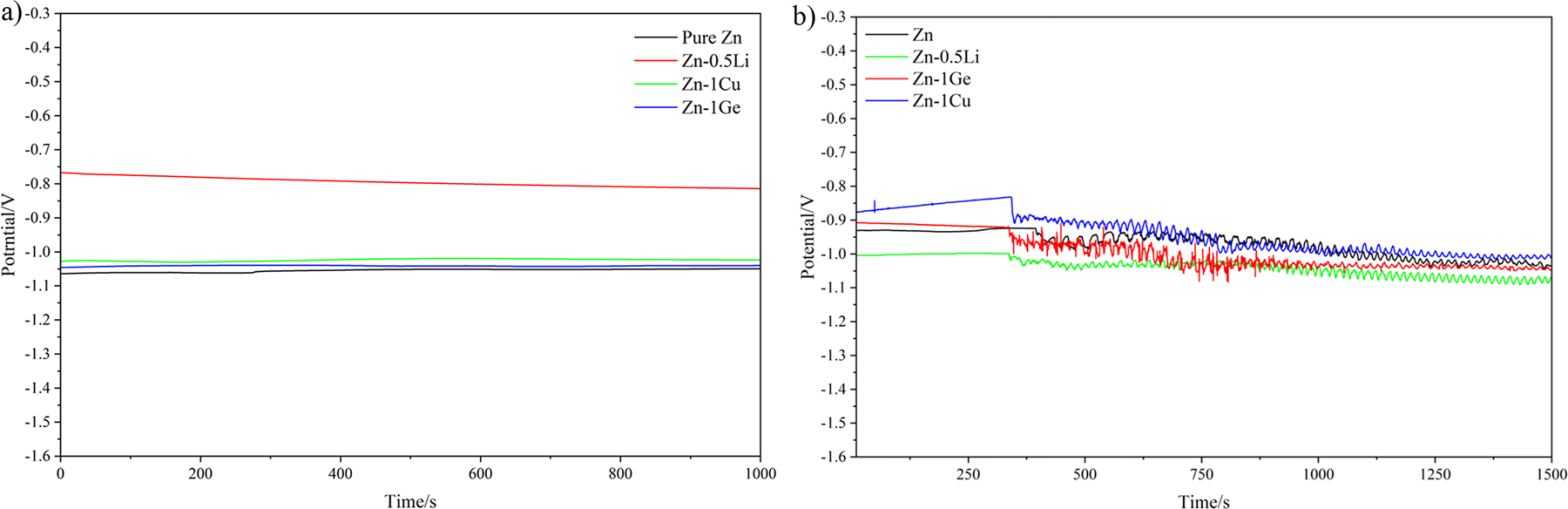
OCP curves of pure Zn, Zn-0.5Li, Zn-1Cu and Zn-1Ge in Hank’s solution: (**a**) without friction and wear; (**b**) with friction and wear.

Figure 8 shows the evolution of open circuit potential (OCP) values of all alloys with friction (Fig. 8a) and without friction (Fig. 8b). Specific values in the figure are given in Table 2. In Hank’s solution, the open circuit potentials of the samples except Zn-0.5Li alloy were not significantly different. The open circuit potential of Zn-0.5Li alloy is higher than that of other samples (See Fig. 8a). It can be seen from Fig. 8b that as the friction and wear experiment progresses, the open circuit potential of the four samples all show a downward trend. And after a certain period of time, the curve suddenly drops and starts to fluctuate. It can be seen from Table 2 that all Zn alloys showed similar open circuit potentials under friction and wear conditions.

**Table 2:** Electrochemical data of pure Zn, Zn-0.5Li, Zn-1Cu and Zn-1Ge.

	With friction and wear	Immersed in Hank’s solution (without friction and wear)	
Sample	Open circuit potential /V	Open circuit potential /V	i_corr_/mA·cm^−2^
Pure Zn	−0.986 ± 0.032	−1.056 ± 0.007	0.0554 ± 0.010
Zn-0.5Li	−0.992 ± 0.027	−0.800 ± 0.013	0.1486 ± 0.008
Zn-1Cu	−0.929 ± 0.015	−1.030 ± 0.008	0.0542 ± 0.007
Zn-1Ge	−0.990 ± 0.042	−1.034 ± 0.005	0.0552 ± 0.005

The open circuit potential values of the samples undergoing friction and wear experiments in Hank’s solution environment were compared with those of the same samples without friction and wear in the same environment. It can be seen that the open circuit potential values of the samples without friction and wear, except Zn-0.5Li alloy, are lower than −1V. The open circuit voltage values of the samples under friction and wear operation are increased, which are all greater than −1V.

**Fig. 9:**
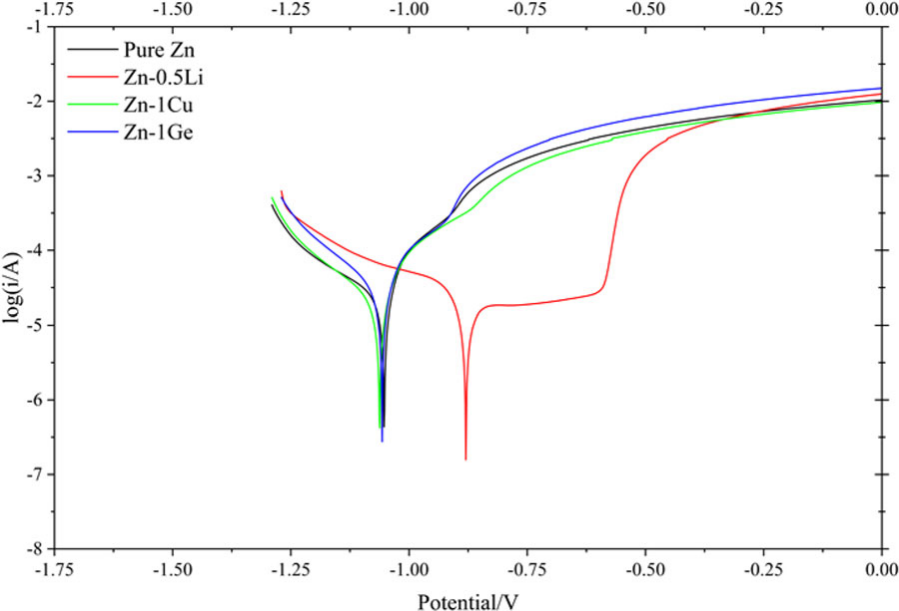
Polarization curves of pure Zn, Zn-0.5Li, Zn-1Cu and Zn-1Ge immersed in Hank’s solution.

Figure 9 shows the polarization curves of the four samples immersed in Hank’s solution. Specific values in the figure are given in Table 2. Except for Zn-0.5Li, the other three samples have similar corrosion potential and corrosion current density. The E_corr_ of the four samples immersed in the simulated body fluids is: Zn-0.5Li > pure Zn > Zn-1Ge > Zn-1Cu. As can be seen from Table 2, the I_corr_ of the four samples is as follows: Zn-0.5Li > pure Zn > Zn-1Ge > Zn-1Cu. The results show that Zn-0.5Li has the best corrosion resistance in Hank’s solution.

## Discussion

It is well known that alloying of second element into the base metal can change the mechanical properties, corrosion behavior and wear behavior of the material. In the present study, the friction and wear behavior of various biodegradable Zn-based alloys with Li, Cu and Ge elements in air and Hank’s solution and the synergistic effect of wear and corrosion on Zn-based alloys were investigated.

### Microstructure characterization of biodegradable Zn-based alloys

It can be seen from the Zn-Ge phase diagram [36] that the solid solubility of Ge in the Zn matrix is 0%, and Ge does not form a solid solution after being dissolved in the Zn matrix. When the Ge content is <5.8 wt.% (Ge content of the eutectic point), the alloy is mainly composed of α-Zn phase and Ge-containing eutectic phase. Therefore, the result of XRD found that Ge simple substance exists in the Zn-1Ge alloy. During the plastic deformation process, the Ge-containing eutectic phase can hinder the movement of grain boundaries, and can also refine the grains, improve the strength and hardness of the Zn-1Ge alloy, thereby improving the wear resistance of the alloy.

It can be seen from the Zn-Li phase diagram [37] that β-LiZn_4_ phase may be produced in the alloy when Li content is 0.5% and non-equilibrium solidification. According to XRD results, it is found that element Li precipitates as a part of eutectic structure in the form of LiZn_4_.

According to the Zn-Cu phase diagram [38], the maximum solid solubility of Cu in Zn matrix is about 1.7wt.% (425 °C). The solution of 1 wt.% Cu in Zn matrix will form η-(Zn) solid solution. Therefore, Zn-1Cu alloy is mainly composed of solid solution and a small amount of ε-CuZn_4_ second phase. Therefore, Cu dissolved in the Zn matrix has a solid solution strengthening effect. The dispersion of CuZn_4_ phase can also hinder the dislocation motion and improve the strength of the material.

### Wear behavior of biodegradable Zn-based alloys

Hank’s solution has two effects on the wear behavior of Zn and its alloys. The simulated body fluid has a lubricating effect on the wear of the material, making the COF of the alloy in Hank’s solution lower than the COF under dry sliding conditions. As far as the coefficient of friction is concerned, the lubricating effect of Hank’s solution helps reduce wear.

On the other hand, Hank’s solution has a corrosive effect on Zn and its alloys. The film formed by corrosion will be damaged by friction, leading to further corrosion, and the flaked wear debris will also cause further wear. However, in this experiment, the wear volume of Zn and its alloys under dry sliding conditions is lower than that under Hank’s solution lubrication conditions, indicating that Hank’s lubrication effect is stronger than its corrosion effect during the friction process.

### Corrosion and tribocorrosion behaviors of biodegradable Zn-based alloys

Both friction and corrosion occur when materials are rubbed and worn in Hank’s simulated solution. There are significant differences in the corrosion and friction corrosion behavior of alloys. There is a synergistic effect between friction and corrosion. The open circuit potential is relatively stable when the alloy does not experience friction. However, after friction is added, the open circuit potential of the alloy (excluding Zn-0.5Li alloy) in Hank’s solution becomes higher, and the corrosion tendency is weakened.

At the beginning, a corrosion product film is formed on the surface of the material, which has a physical barrier effect, thereby hindering the diffusion of ions in the solution to the metal matrix. As the friction progresses, the surface corrosion product film is removed, exposing the exposed metal, causing the metal to corrode. In addition, during sliding, the wear track in the metal gradually becomes larger, the exposed area of the metal increases, and the corrosion of the metal gradually intensifies. After the bare metal is corroded, a passivation film of corrosion products will be produced on the surface. Therefore, the process of passivation-de-passivation-re-passivation is repeated continuously in the Hank’s solution, and the corrosion product film is continuously formed and disappeared. Therefore, as the friction progresses, the open circuit potential of the metal suddenly drops, and the overall trend is downward, and the corrosion tendency becomes greater and greater. And the open circuit potential has been fluctuating. There is a synergistic effect between friction and corrosion.

## Conclusions

The tribological behaviors of biodegradable pure Zn and Zn-based alloys were investigated, and the following conclusions can be drawn.

1. The addition of alloying elements Li and Cu can improve the friction properties of Zn under both simulated body fluid and dry friction conditions. Zn-0.5Li alloy has the lowest friction coefficient and the best wear resistance.
2. Hank’s solution has both lubricating and corrosive effects when the alloy is subjected to friction. The friction coefficients of pure Zn and Zn-0.5Li and Zn-1Cu alloys under simulated body fluid conditions are lower than those under dry friction conditions, indicating that the simulated body fluid has a lubricating effect when the material is subjected to friction. The wear volume of Zn and its alloys under Hank’s lubrication conditions is lower than that under dry friction conditions, indicating that the lubricating effect of Hank’s solution is greater than its corrosive effect.
3. There is a synergistic effect between friction and corrosion. In Hank’s solution, the alloys do not only have a single friction or corrosion behavior, but a tribocorrosion behavior. Friction will promote the progress of corrosion.
